# Assessment of Knowledge, Attitude and Practice on Schistosomiasis in Pujini Shehia, Pemba Island, Tanzania: A Blueprint for Planning Community-based Interventions

**DOI:** 10.24248/eahrj.v8i1.745

**Published:** 2024-03-28

**Authors:** Salma Khamis Rajab, Jared Sylivester Bakuza

**Affiliations:** a Department of Zoology & Wildlife Conservation, University of Dar es Salaam, Dar es Salaam, Tanzania; b Vikunguni Secondary School, P. O. Box 203, Chake Chake Pemba, Tanzania; c Department of Biological Sciences, Dar es Salaam University College of Education, Dar es Salaam, Tanzania

## Abstract

**Background:**

Lack of insight into the community's knowledge, attitude and practice (KAP) regarding schistosomiasis stands as a significant obstacle in controlling the disease in endemic regions. Understanding communities' KAP is crucial for designing and implementing appropriate disease control measures. The present study was conducted to assess community's KAP on shistosomiasis in Pujini, Pemba.

**Methods:**

A total of 328 respondents aged 7 to 79 years were selected from schools and the general community using systematic random sampling method. Data collection was conducted using questionnaires, face-to-face interviews and Focus Group Discussion (FGD) to capture communities' KAP and personal experiences and participants' demographic characteristics.

**Results:**

Most participants demonstrated awareness of schistosomiasis, including its transmission, symptoms and preventive measures, although they struggled to distinguish between urogenital and intestinal schistosomiasis. The majority displayed positive attitudes toward the disease, yet over half of them (59.1%) believed that the disease could not re-occur after initial treatment. Notably, older people were significantly less knowledgeable than their younger counterparts (ƴ^2^ = 41.982, df = 5, *p = <.05*) while farmers were also significantly more knowledgeable than other occupational groups like fishermen, livestock keepers and house wives (ƴ^2^ = 36.194, df = 4, *p = .003*).

**Conclusion:**

Community's knowledge about schistosomiasis decreased with increasing age likely due to low levels of education among adults and their poor attendance to health education meetings and campaigns. Despite positive attitudes and awareness toward schistosomiasis, a significant portion of the population continue to be engaged in risky activities such as water contact and poor sanitation practices. Efforts to enhance knowledge, foster positive attitudes, and encourage good practices remains crucial for the successful control and eventual elimination of schistosomiasis.

## BACKGROUND

Schistosomiasis, also known as bilharziasis is caused by trematode worms of genus *Schistosoma*.^1^ The disease occurs in many tropical and sub-tropical countries and is most prevalent in poor communities lacking access to safe and clean drinking water and adequate sanitation.^2^ Schistosomiasis is one of the most serious and prevalent neglected tropical diseases (NTDs) in many developing countries, particularly in Africa, Latin America and Middle East.^3^ Recent estimates show that more than 250 million people are infected with *Schistosoma* species worldwide, and 700 million people are at risk of infection, most of them being from Sub-Saharan Africa.^4^ Tanzania is one of the African countries facing a significant burden of schistosomiasis. Both urogenital and intestinal forms of the disease are present, posing a major public health challenge.^5^

By the year 2012, more than 23 million Tanzanians were infected with schistosomiasis and the country's overall prevalence of the disease at that time stood at 53.3%.^2^ Given the recent increase in the Tanzanian population to 60 million people by 2022^6^, the dynamics of schistosomiasis prevalence are likely to have fluctuated.

Urogenital schistosomiasis is common in Zanzibar (on both Unguja and Pemba islands),^7,8^ where it is transmitted by two species of intermediate host snails; *B. nasutus* and *B. globosus.*^9^ While schistosomiasis is widespread on Pemba Island, it is confined to the north western and central areas of Unguja Island.^7,5^ Patterns of snail distribution and abundance in these areas influence schistosomiasis transmission.^9^

Several control programmes against urogenital schistosomiasis have been implemented in Zanzibar in recent years, particularly on Pemba Island. This has been achieved through provisioning of Praziquantel (PZQ) drugs, health education and community sensitisation campaigns on snail control strategies for prevention and control of the disease.^10,8,11^ These efforts were aimed to enhance the understanding of schistosomiasis among school children, promote behavioural changes within the community, and to reduce contact with water, thereby interrupting the life cycle of the disease.^7^ Thus post-intervention information, particularly regarding the current status of urogenital schistosomiasis-related KAP in Pemba was crucial. Therefore, this study assessed the knowledge, attitudes and practices (KAP) of individuals towards schistosomiasis in Pujini Shehia, Pemba Island. The goal was to generate information that can guide the implementation of integrated community-based prevention and control programmes against the disease in the area as previously suggested.^12^

## METHODS

### Study Design and Setting

This was a cross-sectional study utilising quantitative and qualitative (mixed methods) approaches of data collection. Quantitative methods utilised questionnaires to obtain data on KAP while qualitative methods involved individual interviews and FGD to collect data on individual experiences with schistosomiasis (in the context of KAP). The study was conducted in Pujini Shehia, located in Kusini, Pemba Region on Pemba Island. A shehia is the smallest administrative unit in Zanzibar, equivalent to a Ward (Kata/Mtaa in Swahili) on Tanzania mainland.

**FIGURE 1:**
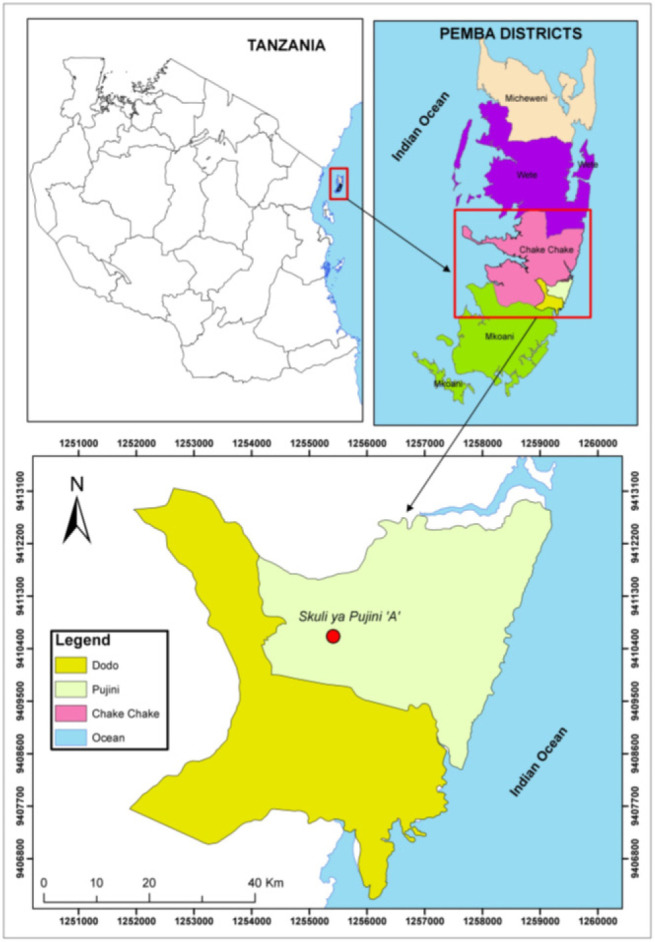
Map of Pemba Island indicating the location of Pujini Shehia where the present study was conducted

**Source:** Tanzania Bureau of Statistics^14^

According to the 2022 census, the total population of Kusini Pemba Region was 271,350 people, of which 139,977 were female and 131,373 were male.^6^ Pujini Shehia is located 10 km from Chake-Chake town (Figure 1). The shehia consists of 6 villages; Kijili, Kibaridi, Mchangani, Mtimbu, Kimbu and Kumvini. It borders 4 other shehias, that is; Mvumoni to the north, Chambani to the south, Mfikiwa to the northwest, Matale to the west, while to the east, it is bordered by the Indian Ocean. (Figure 1). Pujini was selected for the study due to the notable absence of KAP studies on schistosomiasis, despite the area being in close proximity to fresh water streams believed to harbour schistosome-transmitting snails.^13^

### Sample Size Estimation

The study's sample size was obtained using statistical Simple Random Sampling (SRS) formula at 95% confidence limit (the 1.96 value in the formula) as shown below;



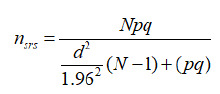



SRS = Simple Random Sampling, n = required sample size, *p* as the estimated proportion in the target population = 50% or 0.5 (recommended when P is unknown), q = 1.0 – p(0.5), N as population size at the time of study (9261 people), d as desired absolute precision (degree of accuracy required) = 0.05. This resulted in a total sample size of 369 participants.

### Participants and Sampling

School children aged 7 to 17 years and adults aged 18 to 79 years were enrolled into the study. For school children, 20 students were selected from each class level (standard one to form four). At each class level, SRS was used to select participants by picking one individual after every fixed or constant interval depending on the number of students in the class. For adult sampling, only one individual was picked from every fourth household in each village.

The study population was first stratified based on school children and community members (adults) while studied villages were chosen randomly from the list of villages in the shehia. Children aged 6 years and below (nursery school level) were excluded from the study because they could not comprehend the consent form or understand the content of the questionnaire.

### Quantitative Data Collection

An approved structured questionnaire was utilised for data collection on KAP. The questionnaire, originally prepared in English was translated to Kiswahili, the commonly spoken language in the study area. Enumerators, were trained before commencing data collection, administered the questionnaires directly to participants. To assess community's knowledge on schistosomiasis, the questions focused on the respondent's understanding of the causes, symptoms, signs, transmission, treatment and prevention of the disease. For gauging attitudes, a combination of open-ended and closed questions were employed. These questions assessed whether schistosomiasis was perceived as harmful or harmless, treatable or untreatable, preventable or non-preventable disease. They also evaluated whether the community considered schistosomiasis an embarrassing desease, if the disease could recur after treatment and the emotional response they would have after witnessing someone with signs and symptoms of the disease.

Regarding practices, participants were questioned about their choices when infected with schistosomiasis, specifically whether they sought medical help from a hospital or a herbalist. Additionally, inquiries were made about the existence of health committees or organisations providing information to the community on the risks of schistosomiasis.

### Qualitative Data Collection

Individual interviews and focus group discussions (FGD) were conducted with purposefully selected participants. Participants in FGD were selected based on specific criteria such as; those sharing the same profession, like teachers and medical personnel. They were grouped together to facilitate and harmonise the discussions as suggested elsewhere.^14,15^ Each FGD lasted between 60 to 90 minutes as recommended.^15^ Open-ended questions were selected purposefully from those used in questionnaire interviews and were applied for both individual interviews and FGDs. Due to logistical challenges, no recording of the discussions was done. Instead, comments and views provided by focus group discussants were directly transcribed as field notes in notebooks.

### Data Analysis

Descriptive statistics were used to determine the frequency of occurrences of KAP parameters in the datasets. Data from individual interviews and FGDs were compiled, analysed and presented as thematic topics based on the responses provided by participants, following standard guidelines.^15^ Data was organised according to common themes, then mapped into framework matrix and analysed as appropriate to make sense of the information, as recommended.^15^ Frequency tables and charts were prepared in MS-Excel 2010. All data was processed and analysed in Statistical Package for the Social Sciences (SPSS) platform, version 19. Chi-square test was used to assess the association of KAP and focus group parameters with demographic and socioeconomic characteristics such as age, sex, level of education and occupation.

### Research Clearance and Ethical Considerations

The research protocol used for the study was reviewed and approved by the Zanzibar Medical Research and Ethics Committee (ZAMREC) via certificate number ST/000/Nov/016. The permission to visit schools was granted by the Ministry of Education while an access to the communities was provided by Chake Chake District Commissioner's Office. On the sampling day, community members were fully informed about the exercise through village leaders.

## RESULTS

### Participants' Knowledge on Schistosomiasis

**FIGURE 2:**
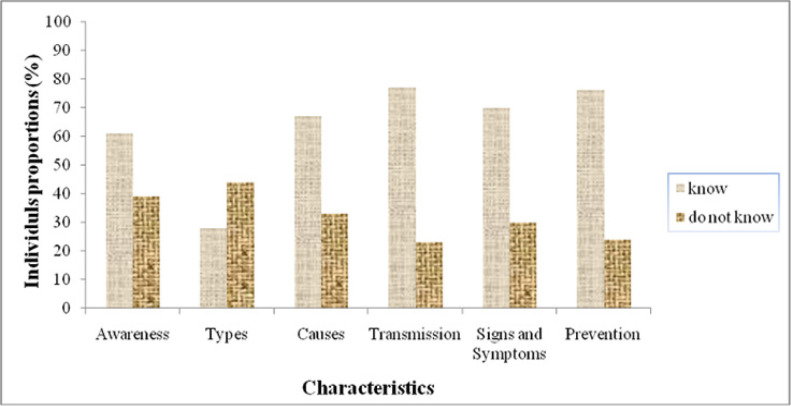
The proportion of individuals with knowledge on various aspects of schistosomiasis in Pujini, Pemba

The study had 328 participants comprising of 205 school children and 123 adults. More than half (61%) of the respondents demonstrated awareness of schistosomiasis. Hospitals, schools, mass media, family and special educational training were identified as sources of knowledge or information on schistosomiasis. Although 53.7% of the participants were aware of urogenital schistosomiasis, none of them was able to differentiate it from intestinal schistosomiasis. Majority of the respondents (over 50%) understood the causes and transmission of schistosomiasis, its signs, symptoms and prevention (Figure 2).

**FIGURE 3:**
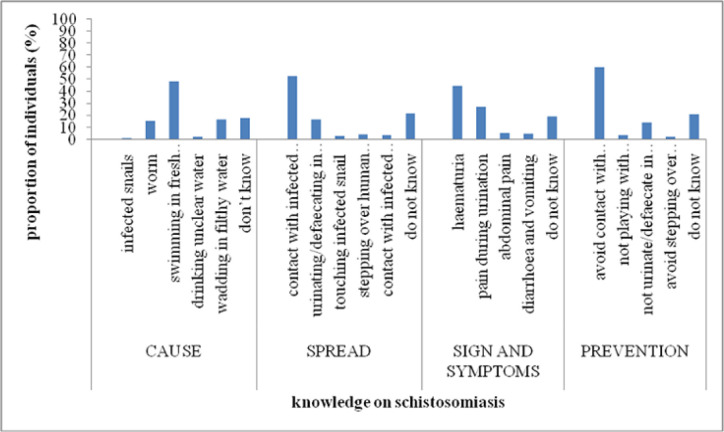
Levels of Community Knowledge on Schisosomiasis in Pujini Shehia

Additionally, more than half of the respondents in Pujini identified swimming in fresh water bodies, contact with infected snails and worms as perceived causes of schistosomiasis. Some participants believed that wading in filthy water and drinking unclean water were potential causes of the disease (Figure 3). Participants also mentioned that contact with cercariae-infested water, urinating and defaecating in fresh water streams could transmit schistosomiasis. However, others thought that stepping over human faeces and urine, engaging in sexual intercourse, or contact with an infected person could lead to acquiring the disease.

Regarding signs and symptoms of schistosomiasis, some participants suggested indicators such as haematuria and pain during urination, while others mentioned diarrhoea, vomiting and abdominal pain. Furthermore, participants mentioned staying away from snail-infested water and avoiding urination in fresh water as potential control strategies for schistosomiasis. Others mentioned avoidance of stepping over human faeces as one of the possible control measures against the disease. Additionally, a percentage of respondents (13.7% - 18.3%) admitted to having no knowledge about the causes, signs/symptoms, transmission and prevention of schistosomiasis.

### Community's Knowledge on Causes of Schistosomiasis

There was no significant association between participants' knowledge and prevalence of schistosomiasis; both knowledgeable and non-knowledgeable individuals were equally infected with the disease (ƴ*^2^ = 3.719, df = 3, p = .293*). Knowledge about schistosomiasis decreased significantly with increasing age with older people being less knowledgeable than the younger ones (ƴ*^2^ = 41.982, df = 5, p = <.05*). Approximately 16.0% of the participants never attended school and were above 40 years old at the time of study. There was no significant variation in levels of knowledge on schistosomiasis between male and female participants (ƴ*^2^ = 0.953, df = 1, p = .329*). In comparison to other villages, the residents of Kumvini village were significantly more knowledgeable about schistosomiasis (ƴ*^2^ = 22.619, df = 5, p = <.05*).

There was no clear pattern or trend between participants' knowledge of schistosomiasis and their education level although, primary school level children were significantly more knowledgeable about the disease than secondary school individuals or adults (ƴ*^2^ = 18.333, df = 2, p = <.05*). Farmers were significantly more knowledgeable than other occupational group such as fishermen, livestock keepers and house wives) (*ƴ^2^ = 36.194, df = 4, p = .003*) (Table 1).

**TABLE 1:** Participants' Knowledge on the Causes of Schistosomiasis in Relation to Demographic and Socio-Economic Characteristics in Pujini Shehia, Pemba Island

Characteristics	Number of respondents (n)	Proportion of individuals with correct knowledge (%)	*p-value*
Age (Years)			<.05
7–8	43	12.8	
9–13	80	31.9	
14–19	83	30.1	
20–39	97	21.5	
40–59	20	4.1	
60–79	5	0.5	
Sex			0.329
Male	166	52.5	
Female	162	47.5	
Village			<0.05
Kumvini	58	23.7	
Kibaridi	65	16.9	
Mtimbu	53	16.4	
Kijili	63	16.0	
Dodo	43	14.6	
Kimbu	46	12.3	
Education			<0.05
None	60	11.9	
Primary education	159	51.9	
Secondary education	109	24.4	
Occupation			<0.05
Farmers	65	12.8	
House wives	32	6.8	
Fishermen	20	5.5	
Livestock keepers	6	1.8	

### People's Awareness on Community Health Committee and Schistosomiasis Control Programme in Pujini

**FIGURE 4:**
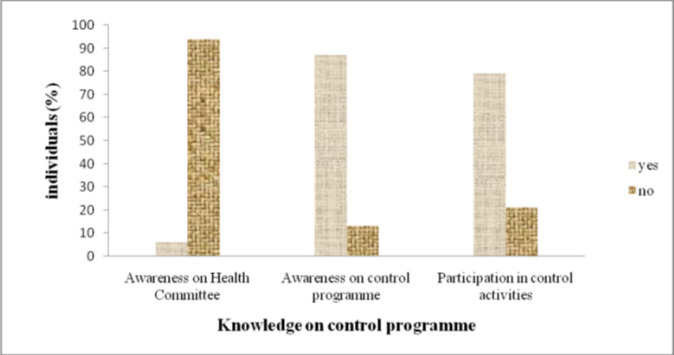
Knowledge on Village Health Committee Activities and Schistosomiasis Intervention Programme in Pujini, Pemba

Most participants (86.6%) demonstrated awareness of the control interventions against schistosomiasis in their villages. Majority of them (76.5%) were able to identify the distribution of PZQ as one of the control approaches being carried out within their villages. Other control approaches mentioned included behavioural change through health education. A significant proportion of respondents (79.3%) actively participated in these activities, while a small percentage (7.6%) expressed unwillingness to participate (Figure 4).

During FGDs, some community leaders mentioned the presence of the community-based health groups in Pujini, with some serving as committee members. In addition, it was revealed that most community members were unaware of the existence of health committees and their functions, particularly school children.

### Drug Provision Challenges from Focus Group Discussion

The school children and the whole community received PZQ and health education twice a year. Drug distribution at school level was carried out by the schools as mentioned by one of the interviewees (School Headmaster) during a FGD;

*I am required to collect drugs from the NTDs office in Pemba in accordance to the total number of students in school. We provide the drugs during the day under the supervision of a medical personnel. This is because, sometimes the drugs may cause dizziness and or itching to students as side effects, hence the medical personnel would handle those problems if they occurred. Some of the students do not come to school intentionally on PZQ distribution days as they are aware of the provision of the drugs, also some students pretend they are taking drugs but hide them under their tongue, do not swallow them and instead spit them out once they go out* (Adult male participants interviewed on 08/10/2016).

Furthermore, a member of the NTDs office in Pemba explained during a FGD:

*The NTDs office in Pemba is responsible for providing drugs to the community under the umbrella of the Ministry of Health (MoH). We hire people temporarily to perform this task. These people are trained specifically for the distribution of the drugs. The distributors pass from household to household and provide drugs to eligible people excluding children less than three years old, pregnant and breast-feeding (lactating) women. The number of PZQ tablets given to each individual depends on their height, which is estimated using the dose pole. There is also an educational programme going parallel to drug provisioning. We provide health lessons pertaining to schistosomiasis such as; risk areas, transmission, symptoms, prevention, treatment, host species (snails), as causative agent, infectious larvae (cercariae) and practices that lead to someone being infected. The target group is school children and madrasa children (Islamic Quran classes) and the community. We use films and verbal (presentation) with the help of pictures and flip charts. After providing them with education, the children are given playing or sports tools that will enable them to concentrate on sports activities and divert their attention from swimming in water bodies* (Adult male discussant, 08/10/2016).

### Community's Attitudes on Schistosomiasis

**FIGURE 5:**
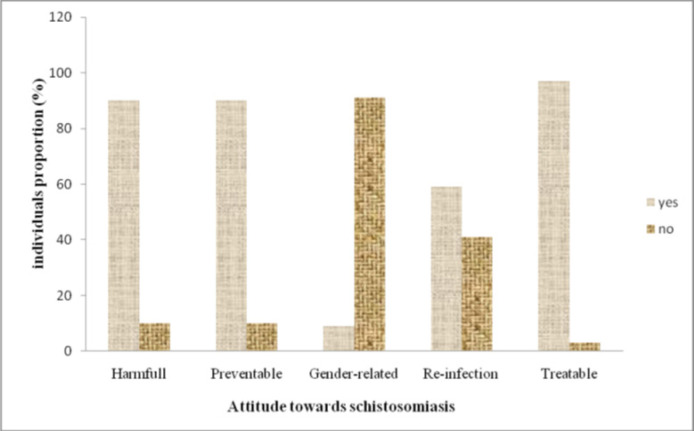
Communities' Attitudes Towards Schistosomiasis in Pujini, Pemba

Some participants regarded schistosomiasis as a relatively benign ailment, asserting that it can be prevented. A significant portion of the respondents (59%) held the belief that schistosomiasis does not reoccur following initial treatment (Figure 5). A vast majority (96.3%) expressed confidence in the curability of the disease through hospital based treatment, with only 3.0% advocating for herbal remedies. Notably, 69.4% of the participants deemed schistosomiasis as a condition entailing shame or embarrassment. In terms of attitude towards individuals affected by schistosomiasis, the survey revealed that 85% of the respondents were willing to provide help. Conversely, 11% expressed an unwillingness to interact with schistosomiasis patients, while 4% admitted to harbouring fears of contracting the disease.

### Community's Beliefs on Schistosomiasis

The majority of respondents perceived schistosomiasis as a harmful condition. A higher proportion of respondents with primary school level education in the age group of 7 to 8 and 9 to 12 years in Kumvini village believed that the disease cannot recur after the first treatment (Table 2).

**TABLE 2:** A Variety of Community's Beliefs on Harmfulness and Reoccurrence of the Disease with Demographic and Socio-Economic Characteristics

Variable	Respondents	Harmfulness of disease		Re-occurrence of disease	
		Harmful %	Not harmful %	Infection recurs %	Infection does not recur %
Age (Years)					
7–8	43	93.0	7.0	27.9	72.1
9–13	80	93.8	6.3	51.3	48.8
14–19	83	86.5	14.5	69.9	30.1
20–39	97	91.8	8.2	68.0	32.0
40–59	20	90.0	10	65.0	35.0
60–79	5	0	0	80.0	20.0
p-value		0.477		<0.05	
Sex					
Male	166	89.8	10.2	62.7	37.3
Female	162	91.4	8.6	55.6	44.4
p-value		0.621		0.191	
Education					
None	60	91.7	8.3	63.7	36.7
Primary education	159	93.1	6.9	50.9	49.1
Secondary education	109	86.2	13.8	68.8	31.2
p-value		0.162		0.011	
Village					
Kumvini	58	89.7	10.3	48.3	51.7
Kijili	63	87.3	12.7	63.5	36.5
Mtimbu	53	98.1	1.9	62.3	37.7
Kibaridi	65	90.8	9.2	58.5	41.5
Kimbu	46	91.3	8.7	56.5	43.5
Dodo	43	86	14.0	67.4	32.6
p-value		0.366		0.427	

### Community's Practices on Schistosomiasis

Out of the respondents already suffering from schistosomiasis, 22% had sought medical attention at the hospital, constituting 27% of the overall respondents. Furthermore, nearly all of the participants (97.3%) were willing to be tested (diagnosed) and treated with PZQ drugs. However, nearly half of the participants preferred to avoid interactions with schistosomiasis patients. An examination of the association between practice and various demographic and socioeconomic characteristics revealed notable patterns. Men were more willing to visit the hospital when sick compared to women (ƴ*^2^ = 19.949; df = 3, p = <.05*) while adults visited the hospital more frequently than school children (ƴ*^2^ = 19.373; df = 3, p = <.05*). Those who perceived schistosomiasis as either harmful or not showed an equal likelihood of seeking medical attention after falling ill (ƴ*^2^ = 3.384; df = 3, p = .336*). In addition, individuals in the 20 to 39 age group visited the hospital more frequently than other age cohorts (ƴ*^2^ = 43.362; df = 15, p = <.05*). Noteworthy, a high proportion of respondents from Mtimbu and Kibaridi villages exhibited a higher frequency of hospital visits compared to residents of other villages (ƴ*^2^ = 45.103; df = 15, p = <0. 05*; Table 3).

**TABLE 3:** Association Between Community's Practices on Schistosomiasis and Selected Demographic Characteristics

Variable	No of Respondents	Action taken by schistosomisis patients			Not suffering from disease %
		Hospital visit (%)	Herbal visit (%)	None (%)	
Age Group					
7–8	43	18.6	0	22.2	11.8
9–13	80	11.4	41.7	22.2	28.3
14–19	83	1.4	8.3	33.3	30.0
20–39	97	52.9	33.3	33.3	22.4
40–59	20	4.3	16.7	11.1	5.9
60–79	5	1.4	0	0	1.7
Village					
Kumvini	58	12.1	3.4	3.4	81.0
Kijili	63	17.5	1.6	0	81.0
Mtimbu	53	41.5	9.4	3.7	43.4
Kibaridi	65	32.3	3.1	0	64.6
Kimbu	46	10.9	2.2	6.5	80.4
Dodo	43	9.3	2.3	2.3	86.0
Sex					
Male	166	29.5	5.4	3.6	61.4
Female	162	13.0	1.9	1.9	83.3
Respondents age					
School children	205	14.1	2.9	2.4	80.5
Adults	123	33.3	4.9	3.3	58.5

## DISCUSSION

Participants in this study included both school children and adults from Pujini Shehia offering diverse perspectives on their encounters/experiences with schistosomiasis. While the majority of participants could provide insights into various aspects of schistosomiasis such as; transmission, signs/symptoms and preventive measures, a notable gap existed in their ability to discern the similarities or differences between the different types of schistosomiasis as a disease. Consequently, the experiences and viewpoints of study participants highlight the levels of community knowledge, attitudes and practices regarding schistosomiasis among residents of Pujini Shehia on Pemba island.

Baseline studies before the onset of randomised intervention trials in Pemba revealed the presence of poor community knowledge on schistosomiasis transmission and prevention.^9^ It was also concluded during the study that, farming, fishing and swimming contributed to the risk of schistosomiasis transmission on the island.^12^ Moreover, the perception of the community on schistosomiasis was contradictory, as for example, some of the people considered the disease as a female infection while others thought it affected male individuals only.^11^ Furthermore, some of the people thought that witchcraft and sexual intercourse were among the means through which schistosomiasis was transmitted.^11^ Thus, the present study's findings on people's KAP in Pujini Shehia provide essential data for assessing recent control programmes and planning and implementation of future control strategies against schistosomiasis.

### Participants' Knowledge on Schistosomiasis

In this context, knowledge refers to individuals' awareness on schistosomiasis.^16^ More than half of the respondents demonstrated some level of familiarity with the disease, although majority struggled to differentiate between urogenital and intestinal schistosomiasis. Notably, this study was carried out in an area with active control interventions, potentially contributing to the high community awareness observed. These findings are in agreement with previous studies' findings in Senegal and China. ^12,17^

The study's findings reveal that hospitals and schools play pivotal roles as primary sources of information on schistosomiasis, whereas the contribution from mass media and family is significantly low. The results support the need to strengthen the role of teachers in health education. One potential avenue for enhancement is the introduction of extra curriculum activities focusing on environmental health and personal hygiene. Additionally, there is need to emphasise the importance of community or village meetings as platforms for discussing public health matters and delivering essential health education messages, aligning with recommended strategies.^16^ Furthermore, the study suggests the integration of both school and community into health education programmes so that children can act as agents for the diffusion of health education messages.^18^ The importance of school as a key source of information for schistosomiasis has also been reported in studies conducted in Brazil.^19^ Thus, recognising the importance of schools as key information sources, the study advocates for the active involvement of diverse channels such as mass media, non-governmental organizations (NGOs), and religious leaders in disseminating health education on schistosomiasis control. This multifaceted engagement is deemed crucial for comprehensive and effective health education outreach.

Majority of individuals in the study area were not aware of the life cycle of schistosomiasis as they were unable to explain the role of snails in the transmission of the disease. However, more than half of the participants were familiar with urogenital schistosomiasis, only few had knowledge about intestinal schistosomiasis, and none could differentiate the two types of the disease. Most people in this area believed that snails are dropped in fresh water bodies (rivers or lakes) during rainy season and many regarded these organisms as harmless, even suggesting that they could be handled or played with without concern.

As highlighted above, most people in the study area were more conversant with urogenital schistosomiasis than intestinal schistosomiasis, which probably is due to the fact that the former is the only type of schistosome-caused disease present in Zanzibar.^11,13^ The health education provided to the community was mainly about urogenital schistosomiasis and its control. While residents of Pujini possess substantial knowledge about various aspects of schistosomiasis, some misconceptions were noted. For instance, attributing the disease to wading in filthy water, drinking unclean water, stepping over human faeces or urine and contact with infected individuals were among the perceived causes of schistosomiasis. Additionally, symptoms such as diarrhoea, vomiting and abdominal pain were stated as the symptoms of the disease, reflecting misunderstanding about the disease. To rectify these misconceptions, sustaining health education efforts is important to augment existing knowledge and dispel inaccuracies surrounding schistosomiasis.

The study's findings also reveal a correlation between decreasing community knowledge about schistosomiasis and increasing age (Table 1). This trend may be linked to lower overall level of education among adults, or due to their poor attendance at health education meetings and campaigns resulting from engagement in various socio-economic activities such as; farming and fishing. Notably, knowledge on schistosomiasis was significantly associated with education as primary school children had better understanding of the disease compared to adults. School children especially those aged 7 to 13 years were probably better in grasping new knowledge or information compared to adults. Furthermore, a notable relationship was observed concerning occupation, with farmers demonstrating better understanding of schistosomiasis than other occupational categories.

Most participants were also aware of the ongoing schistosomiasis control programmes in Pemba. Although they did not understand the details of the project, most of them were involved in the control activities. Consequently, these systematic control interventions not only enhance awareness levels but also foster willingness to participate within the communities. This is evidenced by the high participation observed among infected individuals who sought counselling and treatment against schistosomiasis at health centres.

Similar to the decreasing trend in knowledge about schistosomiasis with increasing age, individual participation in schistosomiasis control activities was significantly associated with age. The level of participation was inversely related to participant's age whereby young-aged individuals mostly school children were predominantly involved in the intervention programmes. This inclination is likely attributed to the ease of managing younger participants during intervention activities. On the other hand, only a modest 6.4% of the study participants were aware of the existence of the village health committees. This suggests potential inefficiencies in the committee's operations or insufficient community sensitisation. Such trends could pose challenges to the effectiveness of the community-based control programmes.

Drug distribution to the community in these areas faces significant challenges, primarily stemming from negative attitude towards the medications. Some individuals harboured suspicions about hidden motives behind the supply of these drugs, with a subset even perceiving the drugs as a birth control mechanism. Other people were reluctant to take the drugs in the absence of a schistosomiasis confirmatory test. This scepticism is evident in various behaviours, such as students avoiding school on the day of drug distribution or attempting to conceal and subsequently discard the drugs. Consequently, these challenges may lead to the persistence of the disease in both adults and school children.

Health education plays an important role in the prevention and control of schistosomiasis as it raises the level of awareness on transmission and spread of the disease. The augmentation of knowledge and awareness levels stimulate positive attitudes and good practice against the disease. However, sometimes, knowledge provided is insufficient to protect people from the infection. This inadequacy stems from the lack of alternative options such as access to alternative water sources and proper sanitation.^20^

### Community's Attitudes on Schistosomiasis

Attitudes in this context and as defined by other researchers,^16^ refers to the manner in which individuals or the public view or perceive a disease that influences their response when they become infected with it. A good attitude, in most cases, reflects possession of correct and appropriate knowledge,^21^ and also indicates the course of action an individual might take in case of disease occurrence. The study revealed that, the majority of participants exhibited a positive attitude toward schistosomiasis. This positive disposition holds promising implications for schistosomiasis control, as it contributes to reducing the rate of disease transmission and re-infection. Therefore, community attitude is an important key in the control of any disease, achieved through the provision of appropriate knowledge concerning the disease in question.^20^

Furthermore, the present study has identified numerous myths and misconceptions surrounding schistosomiasis, particularly regarding its causes and transmission. These findings highlight a lack of community health education, especially as the root causes of schistosomiasis.

Moreover, a significant proportion of respondents exhibited positive attitudes towards schistosomiasis. While some considered it a harmful disease, others believed it was preventable, treatable, non-heritable and capable of infecting both male and females. On the contrary, a minority of respondents held negative views about the disease (Figure 5). This negative attitude poses a challenge to schistosomiasis control and prevention efforts because individuals at risk of contracting the disease or already infected may not perceive the importance of seeking medical attention. Instead they may contribute to environmental contamination, further increasing disease transmission. This underscores the need for future control programmes to incorporate comprehensive health education targeting all relevant demographic groups.

Furthermore, a greater number of respondents believed that, schistosomiasis could be treated in hospitals rather than at herbal clinics. This positive attitude suggests that community members are likely to seek medical care at health facilities immediately or soon after falling ill. Conversely, some studies,^22,23^ reported negative attitude toward schitosomiasis, noting perceptions of the disease as shameful in countries such as Tanzania and Nigeria. Similarly, other investigators,^16^ reported the presence of social stigma, which exacerbates feelings of fear and shame, ultimately deterring people from seeking appropriate treatment. Regarding re-infection, the majority of participants in this study were under the impression that schistosomiasis could not recur after treatments. This misconception presents a challenge for both past and future control interventions, as it may lead individuals to engage themselves in risky behaviour or practices, believing they are immune to re-infection. Addressing these attitudes and misconceptions through targeted health education is crucial for the success of schistosomiasis control interventions.

### Practices on Schistosomiasis

Practice encompasses approaches taken by an individual or community in an event of infection.^24^ Despite exhibiting positive attitudes and having awareness about the transmission and prevention of schistosomiasis, a significant number of participants in the present study continued to engage themselves in high risk activities such as fishing, swimming, bathing and farming in swampy areas.

To mitigate these risks, it is recommended that designated areas for laundry and cattle watering be established. Fishermen and farmers should be provided with sufficient health education on how to protect themselves from schistosomiasis. They should also be advised to visit the hospital regularly for health check-up. Children require parental guidance and close supervision so as to foster behavioural changes that reduce their risk of exposure. The lack of comprehensive knowledge about schistosomiasis perpetuates risky behaviour and practices, underlining the importance of educational interventions. Schools have been highlighted as critical venues for educating children about schistosomiasis although changing human behaviour remains a challenging endavour.^25^

With respect to treatment-seeking behaviour, individuals who had previously suffered from schistosomiasis exhibited high levels of awareness. This heightened awareness often stemmed from their experiences in seeking treatment at health centres, though some admitted to have sought treatment from herbal clinics rather than hospitals. Reliance on herbal treatment can have negative implications toward the transmission and control interventions of schistosomiasis as such remedies cannot kill the young worms,^26^ leading to persistence of the disease.

### Strengths and Limitations of the Study

One of the major strengths of this study lies in its rural setting, characterised by limited accessibility. The study has generated baseline information on the KAPs concerning schistosomiasis in less studied areas of Pujini, Pemba. This information is invaluable in planning and implementation of community-based control measures against the disease. On the other hand, the major weakness of this study is its lack of a tool to establish, through KAP studies, how communities can be assisted to improve their understanding and avoidance of schistosomiasis risk factors. Thus, future KAP studies should explore how public health education can be effectively administered to assist the study population in adopting preventive measures against schistosomiasis. Another limitation of this study is the exclusion of children in interviews and questionnaires assessment instead of focusing on their caregivers, parents or guardians, which may have resulted in missing key data, given their inability to provide consent to participate. Nevertheless, the direct participation of children in the study offered insight into their own views (KAPs), which have the potential to influence plans and decisions in schools and communities for schistosomiasis prevention and control processes.

## CONCLUSION

There is presence of good knowledge and practices as well as positive attitudes on schistosomiasis among the people of Pujini Shehia on Pemba Island Tanzania and notable community participation in intervention programmes. Pockets of low KAP among some residents in the area that may be associated with a number of risk factors responsible for transmission of the disease in the area. As such, well-designed public health education programmes should continue to be administered in the community to improve their understanding and avoidance of risk factors for the disease. Community involvement in drug provision and health education is very important to bring a sense of ownership which can enhance the sustainability of the intervention. So, providing efficient health education to people residing in schistosomiasis-endemic areas is essential for effective and sustainable control programmes against the disease.
